# Three-dimensional simulation of aesthetic outcome from breast-conserving surgery compared with viewing photographs or standard care: randomized clinical trial

**DOI:** 10.1093/bjs/znab217

**Published:** 2021-08-09

**Authors:** A R Godden, A Micha, L M Wolf, C Pitches, P A Barry, A A Khan, K D C Krupa, A M Kirby, J E Rusby

**Affiliations:** Department of Breast Surgery, Royal Marsden NHS Foundation Trust, Sutton, Surrey, UK; Independent patient co-designer, Institute of Cancer Research, Sutton, Surrey, UK; Department of Breast Surgery, Royal Marsden NHS Foundation Trust, Sutton, Surrey, UK; Department of Breast Surgery, Royal Marsden NHS Foundation Trust, Sutton, Surrey, UK; Independent patient co-designer, Institute of Cancer Research, Sutton, Surrey, UK; Department of Breast Surgery, Royal Marsden NHS Foundation Trust, Sutton, Surrey, UK; Department of Plastic Surgery, The Royal Marsden NHS Foundation Trust, London, UK; Department of Breast Surgery, Royal Marsden NHS Foundation Trust, Sutton, Surrey, UK; Department of Breast Surgery, Royal Marsden NHS Foundation Trust, Sutton, Surrey, UK; Independent patient co-designer, Institute of Cancer Research, Sutton, Surrey, UK; Department of Breast Surgery, Royal Marsden NHS Foundation Trust, Sutton, Surrey, UK; Independent patient co-designer, Institute of Cancer Research, Sutton, Surrey, UK

## Abstract

**Introduction:**

Over half of women with surgically managed breast cancer in the UK undergo breast-conserving treatment (BCT). While photographs are shown prior to reconstructive surgery or complex oncoplastic procedures, standard practice prior to breast conservation is to simply describe the likely aesthetic changes. Patients have expressed the desire for more personalized information about likely appearance after surgery. The hypothesis was that viewing a three-dimensional (3D) simulation improves patients’ confidence in knowing their likely aesthetic outcome after surgery.

**Methods:**

A randomized, controlled trial of 117 women planning unilateral BCT was undertaken. The randomization was three-way: standard of care (verbal description alone, control group), viewing two-dimensional (2D) photographs, or viewing a 3D simulation before surgery. The primary endpoint was the comparison between groups’ median answer on a visual analogue scale (VAS) for the question administered before surgery: ‘How confident are you that you know how your breasts are likely to look after treatment?’

**Results:**

The median VAS in the control group was 5.2 (i.q.r. 2.6–7.8); 8.0 (i.q.r. 5.7–8.7) for 2D photography, and 8.9 (i.q.r. 8.2–9.5) for 3D simulation. There was a significant difference between groups (*P* < 0.010) with post-hoc pairwise comparisons demonstrating a statistically significant difference between 3D simulation and both standard care and viewing 2D photographs (*P* < 0.010 and *P* = 0.012, respectively).

**Conclusion:**

This RCT has demonstrated that women who viewed an individualized 3D simulation of likely aesthetic outcome for BCT were more confident going into surgery than those who received standard care or who were shown 2D photographs of other women. The impact on longer-term satisfaction with outcome remains to be determined.

Registration number: NCT03250260 (http://www.clinicaltrials.gov).

## Teaser

Aesthetic outcome following breast cancer surgery has well described influence on long-term quality of life. The authors have developed a three-dimensional simulation to model aesthetic outcome following breast**-**conserving treatment and tested it against the current standard of care (verbal description of likely aesthetic outcome) and the viewing of two-dimensional photographs of other women, in a randomized controlled trial. Simulation provided an efficient way to share information in the preoperative setting and demonstrated superior results with regard to preparing women for their aesthetic outcome compared with current methods.

## Introduction

Breast cancer is a common and emotive diagnosis with 55 176 patients diagnosed in the UK annually (2015–2017)^1^. Of all women with surgically managed breast cancer, over half undergo breast-conserving surgery (BCS), with 28 500 operations performed annually in the UK^2^. The development of oncoplastic techniques and more effective neoadjuvant chemotherapy enable a larger proportion of women to consider breast conservation.

Shared decision making has been a focus of NHS England since 2013 when it took over the Shared Decision-Making Programme from the Quality, Innovation, Productivity and Prevention (QIPP) Right Care Programme^3^. Shared decision making is considered a standard of care in breast cancer. Literature focusing on patients’ experience of shared decision making within breast cancer treatment has described reduced levels of stress, improved knowledge and a preference for a personalized decision-making approach^4^^,^^5^. Shared decision making has recently been adopted by the General Medical Council as a formal recommendation within the decision-making and consent guidelines published in September 2020^6^.

Three-dimensional (3D) simulation is a visual experience that has been used to provide a personalized approach to care. In the cosmetic surgery industry, particularly within breast and facial surgery, 3D surface imaging (3D-SI) and simulation are widely used to facilitate patient decision making^7^^,^^8^. Simulation was reported to be highly reproducible for breast augmentation^9–11^, and proved a useful tool for implant selection^12–15^. Simulation is designed to display complex ideas simply, crossing language and literacy barriers and potentially improving communication in the preoperative planning stage of surgery. 3D simulation may also add value as a tool to improve patient confidence in knowing their likely aesthetic outcome after breast cancer surgery by reducing the gap between patient perception (how they interpret an explanation) and expectation (their visualization of their anticipated result).

The simulation software available uses predefined algorithms to model outcome from aesthetic surgery (implant augmentation, lipo-filling and mastopexy). There is no software currently available to model breast reconstruction or BCS using 3D-SI. Some groups have looked at complex modelling of the outcome of BCS using biomechanics and wound-healing models based on MRI imaging, but these methods involve complex mathematics, are time consuming and expensive, and have not yet been translated to the clinical setting^16–18^.

In many breast units, the standard preoperative preparation for a woman undergoing BCS includes a verbal description of likely aesthetic changes. Often women undergoing breast reconstruction are shown photographs of other women who have had similar operations. Women themselves have explained that looking at other women’s postoperative photographs did not always give them a sense of how *they* would look, and some reported that it felt inappropriate and awkward. The concept of using simulation as part of a preoperative discussion within breast cancer surgery was generated by a patient steering group as a desirable area of study.

The hypothesis was that confidence approaching a BCS operation may be increased if a woman has reviewed simulated images of her own appearance, and this may translate to better satisfaction with outcome in terms of satisfaction with the breasts. Conversely, if the simulation gives a woman an artificially high level of expectation then she may be more disappointed than had she not seen it.

The aim of this study was to establish, using a randomized controlled trial design, which preoperative intervention best prepares women for their likely aesthetic outcome after BCS: verbal description alone; viewing others’ (2D) photographs; or viewing a 3D simulation of their own likely appearance.

## Methods

### Patient selection

This study received NHS Research Ethics Committee and Health Research Authority approval (clinicaltrials.gov identifier NCT03250260). The Royal Marsden Sutton site, where recruitment took place, predominantly serves the local population with symptomatic and screen-detected breast cancer, rather than providing tertiary care for complex cases. Women with early breast cancer without previous breast surgery undergoing unilateral BCT (wide local excision or therapeutic mammoplasty with the expectation of adjuvant radiotherapy) without preoperative expectation of subsequent contralateral symmetrization were eligible.

###  

Potential participants were identified prior to surgery by the clinical team of four consultant oncoplastic breast surgeons. The study was introduced by a member of the clinical team (either breast care nurse or the surgeon during consultation) and the patient information sheet was issued along with an infographic. A telephone call followed from one of two clinical research registrars, and an initial study consultation was arranged (to coincide with preoperative assessment to limit the number of hospital visits). During the recruitment process, a standardized explanation of the study was given to ensure that patients understood that the researchers had equipoise.

###  

Participants were randomized before surgery by the Institute of Cancer Research Clinical Trials Unit. This was separate from the recruitment pathway, ensuring allocation concealment. Stratified randomization with random permuted blocks was used for this trial. The mechanism used to generate the randomization lists was a central, independent, bespoke computer program. Patients were randomized into one of three groups with a 1 : 1 : 1 ratio: standard care (verbal description of likely aesthetic changes alone, the control group); viewing 2D photographs of other women who had undergone similar treatment (surgery and radiotherapy) in the past (matched for age, BMI, breast volume and tumour location); or viewing a real-time simulation of an average outcome from BCS using their own 3D-SI. Randomization was stratified by BMI, intention to undergo axillary lymph node dissection and operation type (standard wide local excision or more complex tissue rearrangement). Axillary lymph node dissection and high BMI have previously been shown to be independently associated with poorer patient-reported satisfaction with breasts^19^.

The primary endpoint was the difference between groups’ median score on a visual analogue scale (VAS) administered before surgery for the question:

‘How confident are you that you know how your breasts are likely to look after treatment?’

It was not possible to blind the investigator to the allocated group at the initial study consultation. However, to reduce bias, the VASs were measured *en masse* at completion of recruitment by one investigator blinded to the randomization group.

### Consultation process

One of two investigators (A.R.G. or A.M.) conducted the initial study consultation. Written informed consent was obtained. All women completed the baseline BREAST-Q BCS questionnaire^20^, a validated patient-reported outcome tool and underwent 3D image capture (VECTRA XT®, Canfield Scientific, New Jersey, USA). Vectra® is a 3D photographic image capture system. Six mounted cameras take simultaneous images, which are then integrated into a 3D image viewable on a workstation. The VECTRA XT is easy to use, has a fast capture speed (3.5 ms) and processing speed (80 seconds) and does not require an experienced photographer. Other 3D surface-imaging systems are available. Women in the 2D and 3D groups then reviewed images, either standardized 2D postoperative photographs of other women or their own 3D simulations, respectively. A standardized verbal description was used with all groups to explain likely aesthetic changes in an attempt to equalize the amount of time spent with a member of the clinical team and reduce the possibility of bias. The final part of the initial consultation for all women was to mark the VAS that was administered in a private room and handed to the investigator in a sealed envelope, marked only with the participant’s study number.

### Secondary endpoints

Follow-up is on-going and secondary endpoints will be reported in due course. Here, they are summarized for context. The secondary aims are to investigate the effectiveness of the simulation compared with the other preparatory methods (randomization groups) using a second VAS administered at 3 and 12 months after BCS to address the question: ‘How well do you think the information about how your breasts are likely to look after surgery (discussion, 2D photographs, or 3D simulation) reflects how they actually look today?’ The accuracy of the simulation compared with reality (at 1 year after BCS) will be compared using both linear and 3D measures obtained from 3D-SI. Changes over time with regard to both patient-reported outcome measures (BREAST-Q BCS module) and objective measures taken from annual 3D-SIs will be reported out to 5 years after completion of BCS. The study will end when the final patient completes 5 years of follow-up.

### Simulation model

**Fig. 1:**
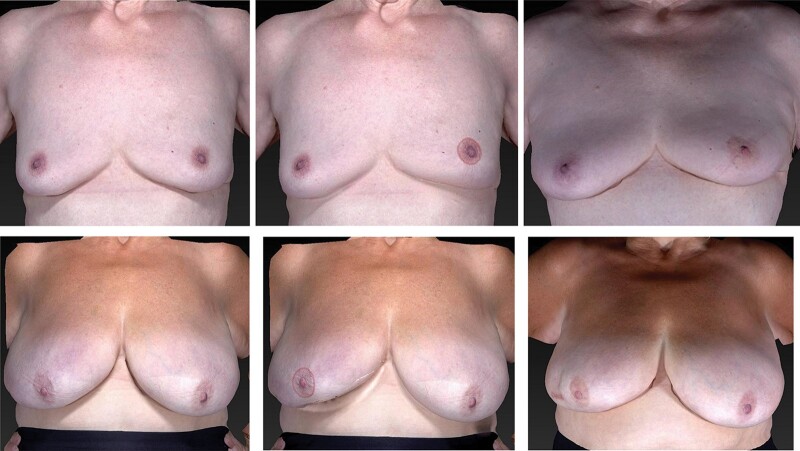
Example simulations Top row (participant A) from left to right; preoperative appearance, simulation of postoperative appearance (left breast), actual postoperative appearance at 12 months after treatment. Bottom row (participant B) from left to right; preoperative appearance, simulation of postoperative appearance (right breast), actual appearance at 12 months after treatment

The simulation method was based on pre-existing software for simulating mastopexy outcome within Mirror™ software, and the VECTRA XT® capture. An adjustment was made to each patient’s preoperative 3D image based upon the average difference for a series of 200 patients who underwent BCS with the best and worst results (65 patients) excluded (135 remained)^21^. Mirror™ software is designed to simulate implant augmentation and mastopexy. Although there are many options for manipulating the images, it is not currently possible to change certain objective measures by a predefined amount. Nipple–sternal notch (N-SN) distance can be manipulated in the mastopexy function, so this was used for the simulation. N-SN distance was reduced by 5 per cent and the nipple moved laterally. A circumareolar scar pattern was used to standardize the type of simulation used. No patient-specific information was entered. Example simulations are shown in *Fig. 1*.

The simulation process was checked using a small number of 3D-SIs from the development series of patients to provide proof of principle: The simulation process was performed on the unoperated breast and compared visually with the treated breast (actual outcome). The accuracy of the simulation to display an average outcome from BCT was deemed acceptable by two reviewers for the purpose of this trial. Natural asymmetry of the breasts is not accounted for in this model, but in the absence of a large set of pre- and postoperative 3D-SI, this was a pragmatic approach.

Prior to showing the simulation to participants randomized to that group it was stressed that the images they were about to see were based on average changes seen in women having similar but not identical treatment, therefore, it was designed to give an idea of the average post-treatment outcome but ‘may not’ represent exactly how they would look. They were told that they may have an aesthetic outcome that is better or worse than the images they were going to view.

### Power calculation and statistical analysis

The primary endpoint was based on a visual analogue scale (VAS). A difference of 1.5 or greater between any two groups, was considered clinically significant. This was based on clinician judgement and feedback from patient representatives. With an estimated standard deviation of 2.0 using a 2-tailed *t* test and a Bonferroni corrected alpha of 0.017 to allow for three comparisons, the study required 39 patients per arm giving a total of 117 patients to have 80 per cent power to detect a difference. Although it was predicted that 15 per cent of patients might be lost due to mastectomy or drop out later, this would occur after completing the VAS and would not, therefore, affect the primary outcome measure and did not need to be accounted for in the power calculation. A Kruskal–Wallis test was used to compare preoperative VAS scores between the three groups with a 5 per cent significance level, with further post-hoc tests to find intergroup statistically significant differences. In presentation of clinicopathological variables, between-group differences were analysed using the one-way ANOVA for continuous variables and the χ^2^ test for categorical variables. BREAST-Q descriptive statics were represented using median (i.q.r.). Between-group differences were analysed using a Kruskal–Wallis test (non-parametric data).

## Results

### Recruitment

**Fig. 2:**
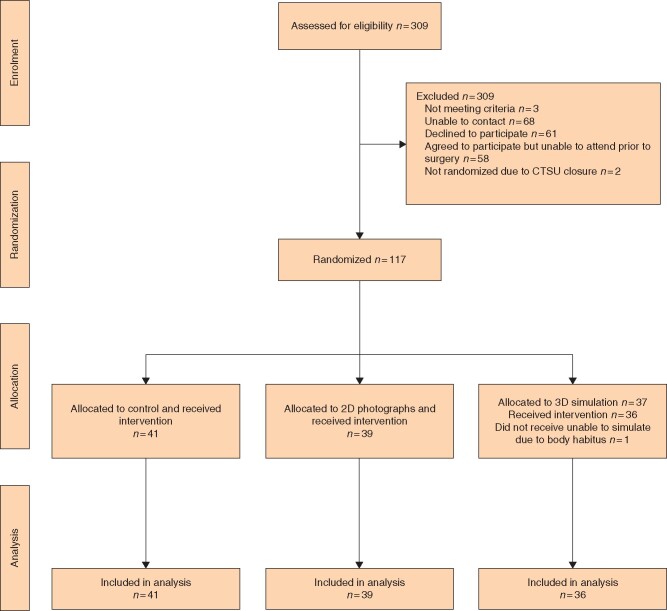
Consort flow diagram This work was in collaboration with the Royal Marsden Clinical Trials Unit, with randomisation services provide by the Institute of Cancer Research Clinical Trials Statistics Unit

The study opened in May 2017 and the final patient was recruited in October 2019. The consort flow diagram is shown in *Fig. 2*. Forty-one participants were allocated to the control group, 39 to the 2D-photograph group and 37 to the 3D simulation group. There were no statistically significant differences in stratification factors by randomization group.

### Demographics

The demographics and clinicopathological data for the study population are shown in *Table 1*. The participants had an average age of 59 years, BMI of 29 kg/m^2^ and were mainly white British, reflecting the local population. The majority of women underwent standard wide local excision (67 per cent) and sentinel lymph node biopsy (79 per cent). Two participants were deemed unfit for surgery after randomization and hence are not included in the histopathological data but are included in the primary endpoint. There were no significant between-group differences for the ‘satisfaction with breasts’ domain of the preoperative BCT BREAST-Q (*P* = 0.343). Group two (2D photographs) was significantly older than groups one (control) and three (simulation) (*P* = 0.013 and *P* < 0.001 respectively). No difference was observed between groups one and three (*P* = 0.777).

**Table 1:** Demographics and clinicopathological data for the simulation study population

Demographics	Total (*n* = 117)	Control (*n* = 41)	2D photographs (*n* = 39)	3D simulation (*n* = 37)	***P***
Age*	59(10)	58(9.17)	64(8.59)	55(10.46)	<0.001
BMI*	29(6)	28(5.65)	29(6.59)	28(5.6)	0.941
**Ethnicity**					
White British	94 (80)	32	31	30	
White other	12 (10)	2	5	5	
Asian	4 (3)	3	1	1	
Black Caribbean	4 (3)	3	0	1	
Mixed white and Asian	1 (1)	0	1	0	
Black African	1 (1)	1	0	0	
Black other	1 (1)	0	1	0	0.426
**Smoking status**					
Non-smoker	61 (52)	19	21	21	
Ex-smoker	42 (36)	16	15	11	
Smoker	14 (12)	6	3	5	0.749
**Clinicopathological information**					
**Laterality of cancer**					
Left	63 (54)	22	20	21	
Right	54 (46)	19	19	16	0.891
**Tumour location**					
Upper outer quadrant	51 (44)	19	14	18	
Upper inner quadrant	17 (15)	6	6	5	
Upper central	12 (10)	5	5	2	
Lateral	11 (9)	1	5	5	
Lower outer quadrant	8 (7)	3	4	1	
Lower inner quadrant	6 (5)	3	3	0	
Lower central	5 (4)	1	2	2	
Central	4 (3)	2	0	2	
Medial	3 (3)	1	0	2	0.091
**Tumour size at diagnosis (primary surgery)**					
Mammogram (mm)*	20(12.89)	22(13.26)	18(13.26)	22(15.3)	0.382
Ultrasound (mm)*	18(11.27)	18(9.49)	20(15.79)	19(9.96)	0.818
MRI (mm)* (*n* = 13)	26(12.34)	31(14.7)	24(2.83)	29(28.44)	0.941
**Surgical information**					
**Type of surgery**					
Wide local excision	78 (67)	27	24	25	
Mammoplasty	39 (33)	14	13	12	0.987
**Axillary surgery**					
SLNB	92 (79)	35	28	29	
ALNC	7 (6)	3	3	1	
None	16 (14)	43	7	6	
TAD	2 (2)	0	1	1	0.618
**Preoperative BCT BREAST-Q**					
Satisfaction with breasts Q-score^†^	64 (53–82)	64 (48–82)	58 (48–71)	58 (53–87)	0.343

Values in parentheses are percentages unless indicated otherwise; Between-group differences were analysed using the one-way ANOVA for continuous variables and the χ2 test for categorical variables.

* values are mean(s.d.),

† values are median (i.q.r.). SLNB, sentinel lymph node biopsy; ALNC, axillary lymph node clearance; TAD, targeted axillary dissection; BCT, breast-conserving treatment.

### Primary endpoint

The total number of participants for the primary endpoint was 116 because it was not possible to perform a simulation using the Mirror™ software for one participant. The final group sizes are uneven due to the number of stratification factors used in the trial. For each possible combination of the factors, there is a separate set of randomized blocks, the aggregated blocks forming a specific ‘randomization list’ for that type of patient. The patients are allocated 1 : 1 : 1 within each list, however, if the number of patients on a list is small the list might not fully complete the blocks of 3 and 6, resulting in a slight imbalance.

**Fig. 3:**
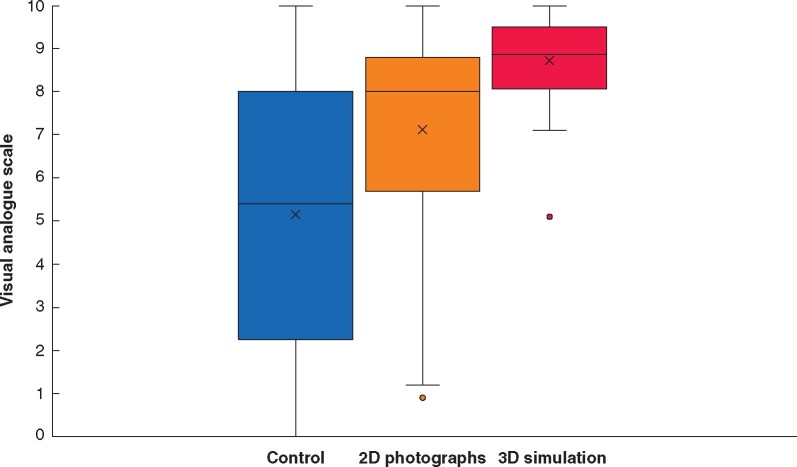
Box and whisker plot demonstrating between-group differences for the primary endpoint VAS in answer to the question administered before surgery: ‘How confident are you that you know how your breasts are likely to look after treatment?’ *n* = 116 (1 failed simulation). X, mean

The median VAS score was 5.2 (i.q.r. 2.6–7.8) for standard care, 8.0 (i.q.r. 5.7–8.7) for viewing 2D photographs and 8.9 (i.q.r. 8.2–9.5) for women who viewed their own simulation. This was statistically significantly higher in the 3D simulation group than for standard care and 2D photograph groups (*P* < 0.001 and *P* = 0.012, respectively). Of note, the women who viewed their simulation showed much less variation in their responses with a tight interquartile range compared with either the control or 2D photograph groups. No statistically significant difference was observed between standard verbal discussion and 2D photographs (*P* = 0.061) although a trend in favour of 2D photographs was noted (*Fig. 3*).

## Discussion

This is the first randomized controlled trial investigating the impact of 3D simulation of appearance on women’s preoperative understanding of aesthetic outcome. This study also evaluates the impact of 3D-SI in the provision of information about BCS. Participants who viewed a real-time 3D simulation of their own likely appearance reported a greater level of confidence in their knowledge of their likely aesthetic outcome than the other two groups.

In the cosmetic surgery industry, particularly within breast and facial surgery, 3D-SI and simulation are widely used to facilitate patient decision making^7^^,^^8^. In a survey of members of the American Academy of Facial Plastic and Reconstructive Surgery, 63 per cent of surgeons already use simulation as part of their rhinoplasty consultation^22^. Patients appreciate the use of simulation in preoperative decision making consultation for aesthetic surgery with a reported 70 per cent of patients undergoing rhinoplasty stating they would decline surgery in its absence^23^. Patients also report a higher satisfaction with 3D simulation over 2D simulation for rhinoplasty^24^. Persing and colleagues used panel evaluation to examine the accuracy of 3D rhinoplasty simulation using VECTRA and deemed actual aesthetic results to be superior to simulation^23^. The group also concluded that experienced surgeons are necessary to translate the simulation into an achievable plan. Simulation was reported to be highly reproducible for breast augmentation^9–11^, and proved a useful tool for implant selection^12–15^. Patients found preoperative simulation for breast augmentation helpful and reported satisfaction with their preoperative decisions^14^. It has been shown to be useful for measuring the anticipated volume changes in aesthetic surgery^9^^,^^13^^,^^25^.

de Runz and co-workers used Crisalix^TM^ (Virtual Aesthetics, Crisalix, Lausanne, Switzerland) to simulate breast augmentation for 38 women. Six months after surgery, 66 per cent of the women absolutely agreed that the simulation represented their actual outcome and 24 per cent partially agreed. Ninety-three per cent felt the simulation helped them choose their implant size, and 97 per cent found the simulation useful^26^. Vorstenbosch and colleagues also used Crisalix to simulate breast augmentation and asked an expert panel to comment upon its accuracy compared with postoperative 3D-SI. The results highlighted baseline breast type as an influencing factor for simulation success^11^. The simulation was deemed to predict overly optimistic results for women with ptotic breasts, and the opposite for women with tuberous breasts. The most accurate simulations were for women with symmetrical breasts at baseline^11^. This may also be relevant with Mirror^TM^ software as it was difficult and occasionally not possible for the software to perform simulation in women with large breasts, high BMI or grade 3 ptosis (Regnault classification)^27^. These studies were concerning breast augmentation where alteration of breast volume is more predictable (a standardized implant) compared with BCS for cancer where the volume (of tumour) excised is rather less exact. Clinically this may still be pertinent to patient selection for simulation for BCS until a workaround with the software can be written or bespoke simulation becomes normality.

The simplicity of the simulation method was one of the strengths of this study. It provides a rapid simulation of aesthetic outcome (less than 5 minutes), not requiring complicated calculations and could be completed during a clinical consultation. This simulation enabled participants to view an average outcome from BCS on their own breast and provides proof-of-principle that viewing simulation was superior to current standards of explanation, prior to investing time and money in development of a bespoke simulation process. The simple simulation method may also be viewed as a weakness of the study. Although for the majority of women, the simulation would have been close to the actual outcome, for a proportion it would have demonstrated a better or worse appearance than the participant went on to receive. In order to provide an average result and avoid an overly optimistic or pessimistic example, the simulation was centre-weighted and based upon a BCS population who had scored 2 or 3 out of 4 (Harvard cosmesis scale) for aesthetic outcome. Inaccuracy of the simulation may influence subsequent patient-reported outcome measures, which will therefore be reported as secondary endpoints.

The development of a bespoke simulation may be required to represent aesthetic outcome better, allowing the surgeon to take individual technical and patient factors into account, such as the size and location of the tumour, scar pattern and potential for symmetrization. The prediction of aesthetic outcome for oncological resections presents additional challenges compared with aesthetic procedures due to the degree of uncertainty with regard to excision volumes and the effect of adjuvant treatment. Simulation may be used as an adjunct to the viewing of 2D photographs in order to give a range of outcomes for patients to avoid setting expectations too high in the initial phases of development.

There is also clinical interest in development of simulation as a tool for shared decision making, as this may help women considering complex oncoplastic breast conservation and breast reconstruction, that is therapeutic mammoplasty with or without symmetrization *versus* unilateral mastectomy and reconstruction or between different types of reconstruction. Knoops and colleagues have developed a machine-learning base framework method using 3D-SI (3DMD, LLC, Atlanta, Georgia, USA) to simulate postoperative appearance for craniofacial surgery with the intended application of more precise surgical planning and outcome evaluation^28^. The use of artificial intelligence may be the most accurate, reliable and efficient way to develop simulation for breast reconstruction, although a large library of images would be required in order to capture the diversity of UK oncoplastic breast surgical practice.

A potential area of bias is that more time spent with a clinician is an intervention in itself. To mitigate against this, although the simulation group was compared with relevant alternatives including a verbal description (control group) and the viewing of 2D photographs, every woman had a 3D image capture at baseline (so experienced the technology even if they did not see their own simulation). The number of investigators was minimized at two, and a standard description was used for every participant, regardless of group, to explain the common aesthetic changes observed from BCS prior to randomization. The investigator and patient could not be blinded during the allocated consultation and the potential psychological influence of ‘getting to see’ their simulation could not be avoided. Nor could the discussion into expected aesthetic outcome between the participant and their surgeon in the clinical environment be controlled, though this happened prior to randomization. The investigators were blinded to the randomization group during outcome analysis. The primary endpoint was the patient’s response on a VAS to a non-validated question about confidence in knowing their likely aesthetic outcome after surgery. There was no alternative validated questionnaire option at the inception of this study.

It is well described that improving preoperative information can positively influence postoperative recovery amongst surgical patients^29–31^. Preoperative counselling in breast cancer patients has been shown to improve social recovery including postoperative aesthetic satisfaction^32^. Involvement in decision making per se, although preferred by many women, is not reported independently to influence psychosocial morbidity, rather the quality of information given seems to be the crucial step in long-term psychosocial adjustment^33^. Within the breast-reconstruction population, improved preoperative education has been reported to reduce decisional regret and improve satisfaction^34–36^.

The superiority of 3D simulation in preoperative explanation of aesthetic results is demonstrated in this study. The utility of simulation for BCS in routine clinical practice has yet to be defined, as has whether managing expectations before surgery using simulation will affect longer-term outcomes such as satisfaction with breasts and, as a result, psychosocial quality of life. With completion of follow-up, the long-term influence of simulating outcome on patients’ ‘satisfaction with breasts’ and quality of life may provide additional rationale for the routine use of simulation in improving the quality of survivorship. The 5-year follow-up has been designed to measure the evolving changes to the post-treatment breast objectively over time. Potential influencing factors such as operation type and progressive fibrosis from radiotherapy will be assessed. This information will be used to refine bespoke simulation and to aid informed preoperative decision making.

In summary, simulation provides an efficient way to share information in the preoperative setting and demonstrates superior results, compared with current methods, when preparing women for their aesthetic outcome. In order to translate simulation from research into actual practice, this needs to be feasible in the clinic. The long-term influence of simulating outcome on patients’ ‘satisfaction with breasts’ and quality of life requires further investigation.

## Notes

This work was presented in part at the Association of Breast Surgery Annual Conference, Glasgow 2019, and was accepted for presentation at The European Breast Cancer Conference, Barcelona 2020.
